# Integration of mapped RNA-Seq reads into automatic training of eukaryotic gene finding algorithm

**DOI:** 10.1093/nar/gku557

**Published:** 2014-07-02

**Authors:** Alexandre Lomsadze, Paul D. Burns, Mark Borodovsky

**Affiliations:** 1 Joint Georgia Tech and Emory Wallace H. Coulter Department of Biomedical Engineering, Atlanta, GA, USA 30332; 2 School of Computational Science and Engineering, Georgia Tech, Atlanta, GA, USA 30332; 3 Department of Bioinformatics, Moscow Institute of Physics and Technology, Moscow, Russia 141700

## Abstract

We present a new approach to automatic training of a eukaryotic *ab initio* gene finding algorithm. With the advent of Next-Generation Sequencing, automatic training has become paramount, allowing genome annotation pipelines to keep pace with the speed of genome sequencing. Earlier we developed GeneMark-ES, currently the only gene finding algorithm for eukaryotic genomes that performs automatic training in unsupervised *ab initio* mode. The new algorithm, GeneMark-ET augments GeneMark-ES with a novel method that integrates RNA-Seq read alignments into the self-training procedure. Use of ‘assembled’ RNA-Seq transcripts is far from trivial; significant error rate of assembly was revealed in recent assessments. We demonstrated in computational experiments that the proposed method of incorporation of ‘unassembled’ RNA-Seq reads improves the accuracy of gene prediction; particularly, for the 1.3 GB genome of *Aedes aegypti* the mean value of prediction Sensitivity and Specificity at the gene level increased over GeneMark-ES by 24.5%. In the current surge of genomic data when the need for accurate sequence annotation is higher than ever, GeneMark-ET will be a valuable addition to the narrow arsenal of automatic gene prediction tools.

## INTRODUCTION

Accurate *ab initio* algorithms are indispensable tools for annotation of eukaryotic genomes since they can identify genes not supported by reliable external evidence (e.g. (1–7)). The predictive power of *ab initio* algorithms is a function of rational algorithm design as well as optimal assignment of species specific parameters. Current Next-Generation Sequencing (NGS) technologies require gene prediction methods that keep pace with the speed of sequencing; *ab initio* algorithms with automatic training offer this advantage. In this paper, we introduce a new approach to automatic training of a eukaryotic *ab initio* gene finding algorithm.

Conventional supervised training techniques are centered on preparation of expert validated training sets; this tedious step now takes more time than genome sequencing itself. Earlier on, the genes compiled into training sets were validated by alignments of the Expressed Sequence Tags (EST) and full length cDNAs sequences. The advent of NGS raised expectations that full length transcripts could be rapidly and confidently produced by assembling RNA-Seq reads. However, assembly of RNA-Seq reads turned out to be non-trivial. The RNA-seq Genome Annotation Assessment Project (RGASP) Consortium (8) comprehensively assessed the accuracy of transcript reconstruction from RNA-Seq data by several algorithms. The results confirmed an informal consensus among developers, that assembly of transcripts from RNA-Seq reads is prone to frequent errors. Thus, mapping assembled transcripts as a fast and accurate approach to gene finding or even to building training sets for *ab initio* gene finders has serious feasibility issues. Consequently, expert control is still necessary in the creation of training sets for the algorithms that employ supervised training.

A supervised gene collection naturally starts from more readily validated genes and is thus likely to be biased towards highly expressed genes, genes with rather long exons, evolutionary conserved ‘core’ genes (9), etc. Unsupervised training (self-training) eliminates expert controlled supervised training.

The major target of unsupervised training procedure is the accumulation (over iterations) of a larger and larger set of correctly predicted genes. Finally, the resulting set of genes serves as the training set for the final estimation of the algorithm parameters. Ideally, this method provides greater ‘ease’ in generating large training sets in comparison with expert controlled accumulation of validated genes. Deviation to an incorrect convergence point is a major risk factor in unsupervised training. It could result in a training set that includes erroneously defined genes or overrepresented subsets of genes of certain type; this kind of issue, however, exists for supervised training as well. A chosen strategy of unsupervised training can be assessed on test sets; notably, the test sets are needed for algorithms using supervised training. Compilation of the test set (relatively small in comparison with the training set) does not present an extra effort specific for unsupervised training.

Identifying the best strategy for unsupervised training is an interesting subject. Earlier we demonstrated feasibility of effective unsupervised training for fungal genomes as well as for compact (<300 Mb) genomes of plants and animals (5,6). Still, we observed that the performance of unsupervised training may degrade for large eukaryotic genomes (>300 Mb in size) that have greater average length of intron and intergenic regions as well as large repeat populations. High genome assembly fragmentation may also present difficulty for unsupervised training.

**Figure 1.**
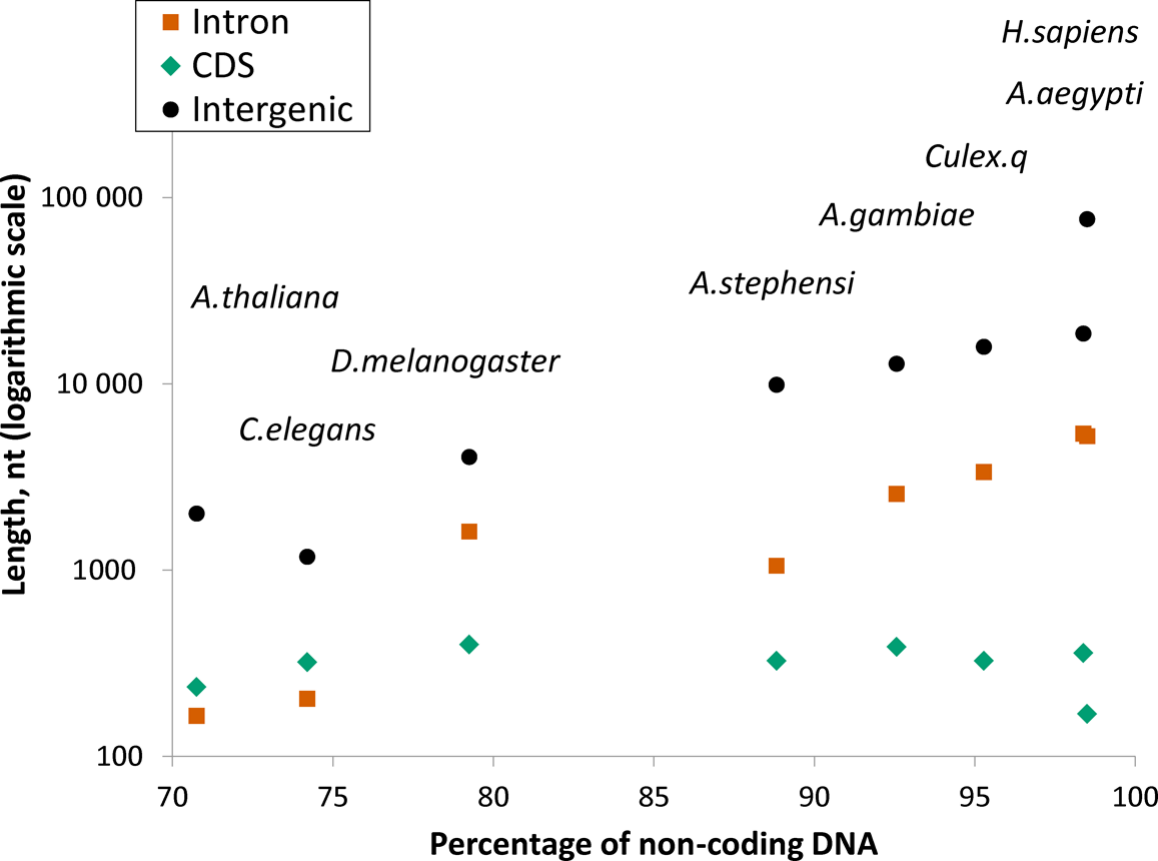
The dot plot graph depicting average lengths of exons, introns and intergenic regions against the value of percentage of non-coding DNA in a given genome was made for the five insect genomes used in the GeneMark-ET tests as well as for several other eukaryotic species. The average lengths of intron and intergenic regions correlate with the genome length while the average length of protein-coding exons (CDS) does not show dependence on the genome size.

In what follows we show how an unsupervised training procedure can use spliced alignments of ‘unassembled’ RNA-Seq reads (rather than assembled transcripts) to improve accuracy of parameter estimation and gene prediction. The key point in combining two independent methods, *ab initio* gene prediction and RNA-Seq read mapping, is introduction of the notion of ‘anchor splice sites’: sites supported by both *ab initio* gene prediction and by RNA-Seq read alignment. Contrary to existing training methods that rely on training sets consisting of complete or almost complete gene structures, the new algorithm uses sets of gene elements, exons and introns, supported by anchor splice sites, to form reliable training sets in the iterative cycles of the model re-training. The new algorithm was tested on genomic and transcriptomic data of several insect species, *Aedes aegypti*, *Anopheles gambiae*, *Anopheles stephensi*, *Culex quinquefasciatus* and *Drosophila melanogaster*, which vary significantly by average size of introns and intergenic regions (Figure 1). We have demonstrated that RNA-Seq support in training boosts GeneMark-ET performance in gene prediction to a higher level in comparison with GeneMark-ES that uses purely unsupervised training. The parameter estimation procedure did show robust performance with respect to variations in the size of the set of mapped introns, repeat content and fragmentation of genome assembly. The new method, used stand-alone or as part of a pipeline, should streamline and accelerate the annotation process in large genomes while improving the accuracy of gene identification.

## MATERIALS AND METHODS

### Data sets

We downloaded from VectorBase (10) sequences and annotations of the genomes of four mosquito species *A. aegypti* (11), *A. gambiae* (12), *A. stephensi* (GenBank: PRJNA167914, submitted by Virginia Tech) and *C. quinquefasciatus* (13). The annotated euchromatin portion of the *D. melanogaster* genome (14) was downloaded from FlyBase (15). Chromosomes 4 and X of *D. melanogaster* and *A. gambiae* were excluded. RNA-Seq datasets for the five species were obtained from the GenBank repository of short reads (16). Characteristics of genome sequences and RNA-seq data sets used in the computational experiments are shown in Table 1.

**Table 1.** Characteristics of the five insect genomes and RNA-seq datasets

Species	Genome sequence						RNA-seq		
	Version	Assembly length (Mb)	Unknown letters (Mb)	Masked seq (Mb)	‘atcg’ seq (Mb)	Number of gaps	Source	Read type	Read count, (millions)
*Aedes aegypti*	AaegL1	1384	74	871	439	36 200	SRR388682	83 nt, single	27.9
*Anopheles gambiae*	AgamP3	273	21	45	207	16 818	SRR520428	85 nt, paired	36.9
*Anopheles stephensi*	AsteV1	208	49	11	148	33 018	SRR643416	84 nt, paired	18.0
*Culex quinquefasciatus*	CpipJ1	528	36	288	204	44 351	SRR364516	50 nt, paired	37.2
*Drosophila melanogaster*	R5	120	0.1	9	111	8	SRR042297	75 nt, paired	13.6

Assembly length includes ‘N’ letters. The ‘atcg’ sequence is the genomic sequence left after masking of repeats. The number of gaps includes gaps with known and unknown length.

To mask repetitive sequence in the mosquito genomes, we used repeat annotations provided by VectorBase (10). To mask repeats in *D. melanogaster* we used RepeatMasker (17) and the *D. melanogaster* genome specific library of repeats from RepBase (18).

The test sets (Table 2) were prepared as follows. In the five insect genomes we selected genes with annotated CDS and 3′ and 5′ UTRs. We excluded particular genes if (i) annotated exons overlapped with masked repeats; (ii) annotated exons (including exons in 3′ and 5′ UTRs) overlapped with other annotated genes; (iii) annotated exons or introns were very short (<6 nt or <20 nt, respectively); (iv) annotated introns were very long (>10 000 nt); (v) annotated exons had read through stop codons, or splice sites had non-canonical (non GT-AG) dinucleotides; (vi) annotated genes had alternative isoforms; (vii) protein products had BLAST hits to the transposable element (TE) protein database; (viii) the best BLAST hit of the protein product to NR database did not have *E*-value better than 0.001. In the tests, the gene prediction programs were run on full genomes, and predictions were compared with annotations of the selected set of genes.

**Table 2.** Numbers of protein coding genes in the genome annotation and in the test set

Species	Annotation version	Number of genes in		Introns in test set
		Annotation	Test set	
*Aedes aegypti*	AaegL1.3	15 998	216	374
*Anopheles gambiae*	AgamP3.6	12 810	420	1061
*Anopheles stephensi*	AsteV1.0	21 785	317	939
*Culex quinquefasciatus*	CpipJ1.3	18 955	360	460
*Drosophila melanogaster*	r5.48	13 842	494	790

### GeneMark-ET algorithm outline

**Figure 2.**
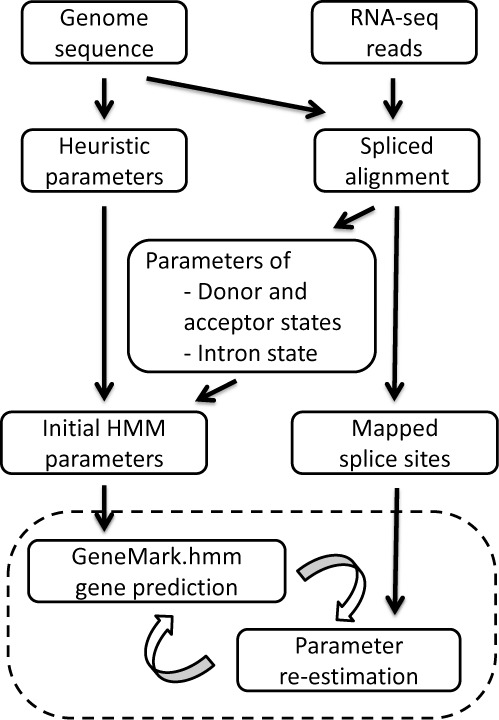
Diagram of the iterative semi-supervised training of GeneMark-ET.

The input data include assembled genomic sequences and RNA-Seq reads as shown in the diagram of GeneMark-ET algorithm (Figure 2). Effectively, the use of mapped RNA-Seq reads, the external (extrinsic) evidence, changes the unsupervised training algorithm GeneMark-ES into an algorithm with semi-supervised training, GeneMark-ET.

At the first step, RNA-Seq reads are aligned to the genomic sequence using a short read alignment algorithm; in our experiments we used several tools, TopHat (19), TrueSight (20) and UnSplicer (21). Only spliced alignments, those that identify *splice junctions* in RNA-Seq reads, the two nucleotides that appear together in mRNA after intron splicing, are of importance for the training algorithm.

**Figure 3.**
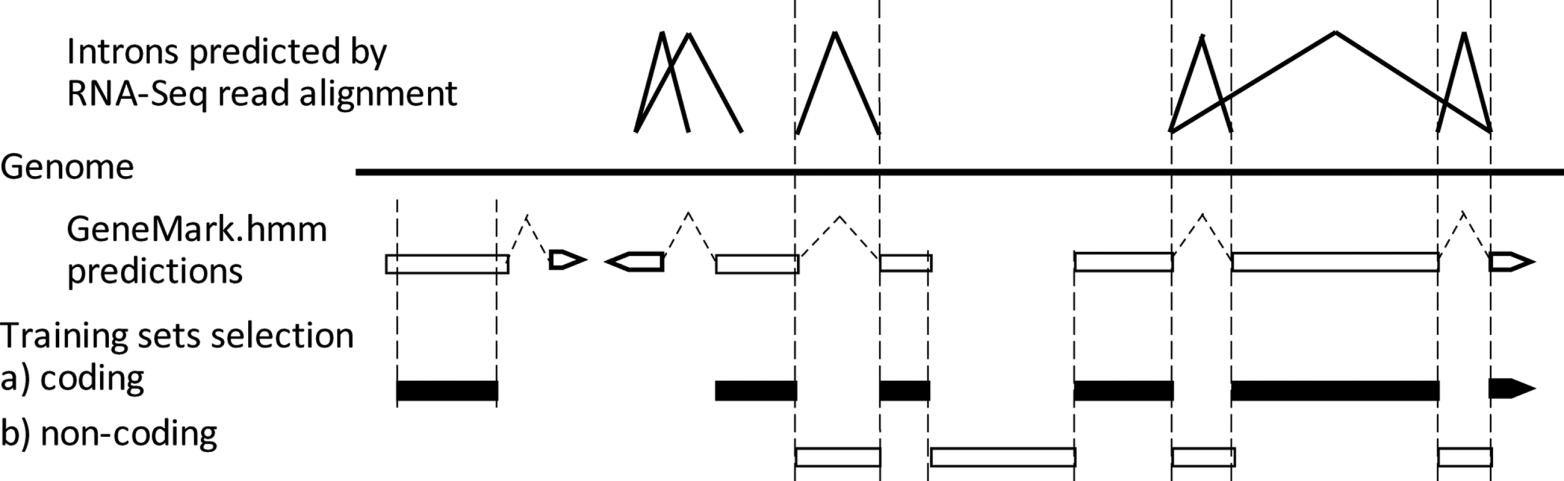
Selection of elements of training set in GeneMark-ET for the next iteration. The new training set of protein-coding regions is comprised from exons with at least one ‘anchored splice site’ as well as long exons predicted *ab initio* (>800 nt).

The general training logic of GeneMark-ET is similar to that of GeneMark-ES (5). First, using an initially defined set of parameters of the hidden semi-Markov model (HSMM) the algorithm predicts protein-coding regions in the chosen genomic sequence. Second, a subset of the newly predicted genes and non-coding regions is selected and used for the HSMM parameter re-estimation. Next, the prediction and re-estimation steps are repeated to convergence. GeneMark-ET differs from GeneMark-ES in the method of selection of the more reliably predicted coding and non-coding regions used for parameter re-estimation. In GeneMark-ET, inclusion of a likely protein-coding exon in the training set requires the predicted exon to have at least one ‘anchor splice site’ (Figure 3). ‘Anchor splice sites’ are those predicted independently by both methods, by the *ab initio* one and by RNA-Seq read alignment.

As an exception to the rule, predicted exons >800 nt (even un-anchored) are selected for estimation of emission probabilities of the HSMM protein-coding states. To account for possible misplacement of a predicted exon boundary, sequences of the ‘long’ exons are trimmed (by 60 nt) on both ends.

Parameters of models of translation initiation and termination site are estimated by using ‘anchored’ initial and terminal exons, respectively.

Parameters of the non-coding region model are derived from sequences of introns having both splice sites *anchored* as well as from predicted intergenic sequences situated between two anchored border (initial or terminal) exons of adjacent genes.

All the HSMM parameters mentioned above are emission probability parameters. Probabilities of transition between hidden states depend less on the anchored sites. Particularly, probability of transition from state of intergenic region to state of the border exon of multiple exon gene is estimated from distribution of the predicted structures of complete genes; this data include structures with both anchored and un-anchored exons.

The first step of training process, assignment of initial algorithm parameters, required special attention. Some parameters could be determined better from the start due to availability of RNA-Seq reads. Among introns mapped by RNA-Seq reads by TrueSight, UnSplicer or TopHat we attempted to identify a subset with likely higher confidence. This subset is used to estimate parameters of the initial models of donor and acceptor sites as well as the intron length distribution.

TrueSight or UnSplicer algorithms assign to a mapped intron a numerical score, *S*, 0 < *S* < 1, with the meaning similar to posterior probability of the predicted splice junction. Introns with scores *S* > 0.5 were selected into the high confidence set. In case of UnSplicer score *S* > 0.5 corresponds to likelihood P < 0.05 that intron mapping is erroneous. In TopHat, the higher is the coverage number (a count of mapped RNA-Seq reads overlapping a predicted splice junction) the higher, in general, is confidence in the predicted splice junction and corresponding intron. When TopHat is the read aligner, the high confidence set includes introns supported by more than three aligned reads.

In the first run of the Viterbi algorithm (Figure 2), we use splice site models and intron length distributions defined from the high confidence set of introns, as well as heuristically derived models of protein-coding and non-coding regions (22); other HSMM parameters are initialized as uninformed priors (5). After the first iteration, the algorithm uses for training the full set of mapped introns, regardless of the probabilistic score (or coverage value).

Similar to the training procedure introduced for GeneMark-ES (5) structures of some sub-models are expanded through iterations, thus, the set of parameters becomes larger. Particularly, each splice site model changes from single frameless models into the set of three phased sub-models related to the three phases of introns used in training. Note that if the predicted *phase* of an intron changes between iterations, a splice site associated with this intron will move from the set of sites with the old phase to the set of sites with the new phase. Also, parameters of the branch point site model, especially important for fungal genomes, are not defined until one of the later iterations (6).

The GeneMark-ET training algorithm stops upon convergence to the set of predicted genes that does not change in further iterations, or upon completing a pre-defined number of iterations.

Accuracy assessment of GeneMark-ET predictions was done on the test sets described above. Sensitivity and Specificity were defined at the levels of splice sites, translation initiation and termination sites, exons and introns, nucleotide and whole gene level. We also added the level of ‘partial genes’, with the meaning of the ‘5′ end incomplete genes’. This level was introduced because a reliable annotation for translation starts is especially difficult to obtain; even in the prokaryotic domain, sets of genes with translation initiation sites validated by N-terminal protein sequencing are scarce. Therefore, computation of 'whole gene' level accuracy that requires exact match of predicted and annotated translation starts may suffer from uncertainty in the translation start position. To calculate Specificity measures, we compared annotation of each gene from the training set to predictions in genomic intervals that include the annotated genes extended by 300 nt to both sides (see ‘Data sets’ section).

## RESULTS

GeneMark-ET was trained on genome sequences and sets of RNA-Seq reads available for the fruit fly *D. melanogaster* and the four mosquito species *A. aegypti*, *A. gambiae*, *A. stephensi* and *C. quinquefasciatus* (see ‘Data sets’ section, Tables 1 and 3).

**Table 3.** Lengths of initial genomic sequence and sequence selected into training process after data pre-processing steps (repeat masking and subsequent filtering of short contigs); sizes of the initial set of introns mapped by RNA-Seq read aligner (UnSplicer) to the full genome and the set of introns mapped to the reduced genome

Species	Genome size (Mb)	Sequence in training (Mb)	Introns mapped to genome	Introns in training	% of introns
*Aedes aegypti*	1384	415	57 684	55 702	96.6
*Anopheles gambiae*	273	201	68 827	59 698	86.7
*Anopheles stephensi*	208	97	28 869	20 418	70.7
*Culex quinquefasciatus*	528	195	57 579	56 621	98.3
*Drosophila melanogaster*	120	97	70 077	56 678	80.9

The 208 Mb *A. stephensi* genomic sequence with 33 018 gaps (four orders of magnitude larger than number of gaps in *D. melanogaster* genome) after masking and filtering short contigs was reduced to 97 Mb (Table 3). The large reduction of *A. stephensi* sequence data was due to the fact that the fragmented assembly contained many short contigs. The *A. aegypti* and *Culex* *q.* genomic sequences with high repeat content were reduced to 415 and 195 Mb, respectively (Table 3). The *A. gambiae* and *D. melanogaster* genomic sequences after reduction were 201 and 97 Mb respectively.

The predictions made by GeneMark-ET on the whole set of genomic sequences were compared with annotation in the locations of the genes included in the test sets (see ‘Data sets’ section). The accuracy of predictions was compared with the accuracy of predictions made by GeneMark-ES. Note that mapping of RNA-Seq reads was not used directly to modify the gene predictions. Thus, improvements in accuracy of GeneMark-ET over GeneMark-ES should be attributed to a more accurate training method delivering better estimates of algorithm parameters.

In our experiments we observed that for all five species, GeneMark-ET outperformed GeneMark-ES. In particular, GeneMark-ET prediction accuracy measured by Sensitivity (Sn or Recall) and Specificity (Sp or Precision) was higher for nearly all elements of gene structure, for whole genes, and for ‘partial genes’.

At the internal exon level GeneMark-ET performed uniformly better than GeneMark-ES. However, the increase in accuracy varied from rather small 0.5% in Sp and 6.0% in Sp for *D. melanogaster* to 22.4% in Sn and 15.4% in Sp for *A. aegypti* (Table 4).

**Table 4.** Assessment of gene prediction accuracy of GeneMark-ES (ES) and GeneMark-ET (ET) gene finders using unsupervised (genomic based) and semi-supervised (genomic and transcriptomic based) training, respectively

		*D. melanogaster*		*A. aegypti*		*A. gambiae*		*A. stephensi*		*Culex q.*	
		ES	ET	ES	ET	ES	ET	ES	ET	ES	ET
Internal exon	Sn	86.7	**87.2**	69.3	**91.7**	77.6	**80.4**	82.7	**85.1**	77.4	**81.8**
	Sp	76.9	**82.9**	60.7	**75.9**	70.3	**78.6**	76.5	**77.0**	54.7	**65.7**
Intron	Sn	82.6	**84.8**	67.9	**89.6**	77.6	**81.0**	85.2	**88.1**	70.2	**81.1**
	Sp	75.3	**79.2**	64.6	**80.3**	73.4	**80.5**	79.4	**81.7**	59.8	**72.7**
Donor site	Sn	85.3	**87.0**	74.6	**92.8**	81.9	**84.1**	88.2	**90.4**	74.3	**83.5**
	Sp	84.5	**86.5**	76.2	**86.8**	82.9	**88.1**	87.3	**88.1**	74.3	**80.7**
Acceptor site	Sn	86.2	**88.2**	74.3	**94.1**	83.0	**86.0**	90.7	**92.8**	83.9	**88.7**
	Sp	85.5	**87.0**	79.0	**89.6**	83.6	**88.9**	87.7	**89.2**	78.0	**84.6**
Initiation site	Sn	71.0	**75.1**	62.5	**79.6**	63.8	**68.1**	65.0	**66.9**	60.8	**76.7**
	Sp	**83.1**	81.5	77.1	**83.9**	**80.0**	79.9	73.6	**76.3**	77.4	**85.7**
Termination site	Sn	77.3	**84.2**	68.1	**88.0**	72.9	**81.0**	83.0	**84.9**	78.9	**82.8**
	Sp	**90.7**	90.0	91.3	**96.0**	89.7	**91.6**	86.5	**92.4**	89.3	**90.9**
Nucleotide	Sn	91.5	**92.1**	87.0	**98.1**	91.4	**92.9**	97.0	**97.3**	93.9	**94.4**
	Sp	**98.3**	97.4	95.2	**96.2**	98.6	**98.8**	98.5	**98.7**	92.0	**93.0**
Gene	Sn	57.9	**63.6**	40.3	**66.7**	43.8	**53.1**	43.2	**48.6**	46.1	**65.0**
	Sp	57.3	**61.0**	42.6	**64.3**	44.0	**53.0**	39.9	**47.0**	44.3	**62.6**
Partial gene	Sn	59.9	**67.2**	41.2	**69.0**	46.2	**56.0**	48.6	**54.3**	48.1	**66.1**
	Sp	59.3	**64.5**	43.6	**66.5**	46.4	**55.8**	44.9	**52.4**	46.1	**63.6**

Bold font highlights the higher accuracy value in a given category and given species. Partial gene level accuracy is computed without taking into account a difference in annotation and prediction of translation starts.

Spliced alignments for GeneMark-ET were produced by UnSplicer.

A similar trend was observed for both whole gene level and ‘partial gene’ levels (Table 4). For *D. melanogaster*, the GeneMark-ET accuracy increased 7.3% in Sn and 5.2% in Sp over GeneMark-ES. For the largest mosquito genome *A. aegypti* the GeneMark-ET predictions improved at the ‘partial gene’ level by 27.8% in Sn and by 22.9% in Sp. For the next largest genome, *C. quinquefasciatus*, the increase numbers were 18.0 and 16.7% respectively, while for the two smaller *Anopheles* genomes the increases at the ‘partial gene’ level were below 10%. The absolute Sn and Sp figures at gene and ‘partial gene’ levels are the lowest for each species (Table 4). These two measures are the most stringent accuracy definitions that require exact prediction of all or almost all the elements of exon–intron structure.

In all the five insect genomes considered here, translation termination sites were predicted more accurately than translation initiation sites by both GeneMark-ES and GeneMark-ET. Difficulty in translation initiation start prediction is related to the presence of alternative in-frame and out-of-frame ATG codons. Also, initial exons are on average shorter than terminal exons and are more difficult to identify. Finally, the annotation of translation starts is likely to be the least reliable element in the annotated exon–intron structure. For instance, most of the translation starts in *D. melanogaster* genome were annotated by ‘the longest ORF in transcript’ rule (http://flybase.org); only a few gene starts were verified by protein N-terminal sequencing.

The accuracy figures for GeneMark-ET shown in Table 4 are the results of algorithm runs utilizing alignments of RNA-Seq reads made by UnSplicer (21). UnSplicer is one of the few methods of choice for RNA-Seq read alignment and splice junction detection. We previously showed (21) that for several species UnSplicer produced a higher accuracy of prediction of splice junctions with respect to genome annotation compared to other methods. This higher accuracy of mapping introns is likely related to reduction in the number of false positives caused by splicing noise and other factors (23,24). However, the results of the GeneMark-ET runs on *D. melanogaster* and *A. gambiae* genomic data with RNA-Seq read mapping done by TopHat or TrueSight (Supplementary Table S1) show that performance of GeneMark-ET is robust with respect to replacing UnSplicer by TopHat or TrueSight. This observation indicates that use of ‘anchored splice sites’ by GeneMark-ET helps filter out instances of false positive splice junction predictions.

To assess dependence of the GeneMark-ET performance on the size of the set of mapped introns we made runs of the new tool on *D. melanogaster* sequence data with mapped intron sets comprising 75, 50 and 25% of the initial set of 70,077 mapped introns (Table 3). Interestingly, the accuracy of gene prediction did not change noticeably in these experiments (data not shown), thus, the algorithm is robust with respect to the changing the number of mapped introns within a wide range. Obviously, if the initial number of mapped introns is too low, GeneMark-ET should be automatically switched to GeneMark-ES mode (current default threshold for the switch is 1000 introns).

**Figure 4.**
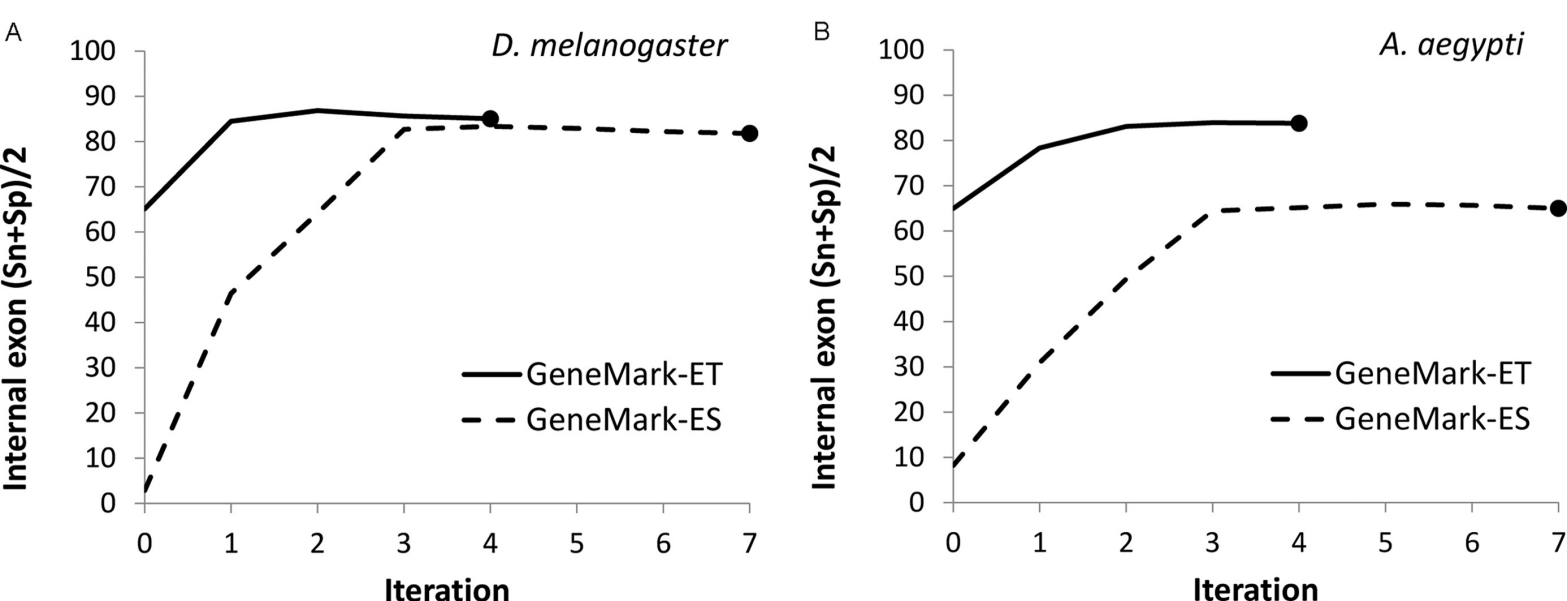
Observed dynamics of change in iterations of the mean of Sn and Sp internal exon prediction values for the GeneMark-ET and GeneMark-ES algorithms in cases of *Drosophila melanogaster* (A) and *Anopheles aegypti* (B) genomes.

We analyzed the dependence of mean values of internal exon Sn and Sp on iteration index for *D. melanogaster* and *A. aegypti* genomes for both GeneMark-ES and GeneMark-ET (Figure 4). The GeneMark-ET initial parameterization integrating information from mapped RNA-Seq reads improved accuracy of predictions in the first iteration by 55–60% in comparison with GeneMark-ES. For *D. melanogaster*, further iterations reduced the large initial gap in accuracy down to 4%. In contrast, for the large *A. aegypti* genome, although the gap was reduced with iterations, the accuracy of GeneMark-ET at convergence remained almost 20% higher than one of GeneMark-ES. Also, GeneMark-ET reached convergence 2–3 iterations earlier (Figure 4). The reduction in number of iterations was observed for the other three genomes as well (data not shown).

The precision of GeneMark-ET in selecting ‘anchored introns’ is illustrated by the following statistics. Only 34 of anchored introns out of 12,554 identified by GeneMark-ET in the first iteration of training do not match *D. melanogaster* annotation version 48 (Supplementary Figure S1).

Information on mapped RNA-Seq reads, besides use in training, could be used directly in prediction steps to make the parse Viterbi algorithm fitting the mapped introns in each iteration. We made an extended version of GeneMark-ET where this approach was implemented in all iterations but the last one. Interestingly, in computational experiments on the *D. melanogaster* genome and the RNA-Seq set this modification did not produce noticeable change in accuracy of predictions on the test set (data not shown).

## DISCUSSION

Annotators of novel eukaryotic genomes frequently use a pipeline, MAKER2 (25) that includes the three gene prediction tools, Augustus, GeneMark-ES and SNAP that independenly generate gene predictions. Training sets compilation for SNAP is aided by mapping of assembled transcripts as well as proteins from protein databases. Alternatively, GeneMark-ES could provide the initial gene models, the lynchpins for the training of SNAP and Augustus. While ‘unsupervised’ training is critically important in this kind of pipeline, many large genomes present difficulties for unsupervised training (e.g. genomes with inhomogeneous composition, or genomes populated with repeats and transposable elements). Assembly of short NGS reads into contigs is a difficult task for genomes with high repeat content. Many published genomes have thousands or even tens of thousands of individual contigs assembled into scaffolds (e.g. mosquito genomes in Table 1). Genome fragmentation creates additional difficulty for algorithm training if the training requires a large set of complete or nearly complete gene structures.

GeneMark-ET presents a new opportunity to deal with the larger genomes. Utilization of filtered RNA-Seq read mapping information improves gene prediction through better algorithm parameter estimation. Effectively, use of RNA-Seq reads along with gene prediction algorithm narrows the training to the genomic regions covered by the transcriptome. GeneMark-ET is designed to use collections of gene structure elements e.g. anchored exons. Training on separate sets of gene elements of similar type increases the volume of sequence in training in comparison with use of whole gene structures. GeneMark-ET is able to complete training even if genome assembly includes a large number of gaps, thus, a large fraction of protein coding genes is likely to be disrupted (as we see for *A. stephensi* genome).

GeneMark-ET was tested on the five insect genomes with sizes ranging from 120 Mb to 1.3 Gb. Insect genomes have relatively few genes in comparison with plant or mammalian genomes of similar sizes, thus the gene density is relatively low. While the accuracy of purely unsupervised training (GeneMark-ES) drops in longer genomes (e.g. internal exons Sn and Sp in Table 4), the accuracy of semi-supervised training (GeneMark-ET) reaches sufficiently high level, comparable to the level reached for compact genomes, such as one of *D. melanogaster.* More specifically, improvement of Sn and Sp at gene and partial gene level is in the range of 18–25% for the largest genomes of *A. aegypti* and *Culex q.*

We should add a general note of caution. Since the test sets were selected to avoid ambiguities (no alternatively spliced genes) as well as to avoid genes with rare features (overlaps, very long introns, non-canonical sites, etc.) the evaluation of performance on such test sets is likely to provide more optimistic figures than would appear in real applications.

Repeat masking is conventionally recognized as a mandatory practical step for improving the accuracy of prediction of ‘host’ genes. However, in a rather rare event, a whole gene of transposable element (TE) or a part of it can be integrated into host genes (26); therefore, rather than brute force masking of all genomic fragment matches to TE library entries, repeat masking prior to gene finding needs a more fine-tuned approach.

The extent to which repetitive sequences may influence the estimation of parameters of gene finding algorithms is an interesting question. The inclusion of protein coding genes encoded in TEs in training sets may lead to biased parameters and to errors in finding true ‘host’ genes (27). For genomes, such as *A. gambiae*, masking repeats before GeneMark-ES training improves accuracy of internal exon recognition by 5%; for larger genomes, like *A. aegypti*, improvement is as large as 15% (data not shown).

Estimation of some GeneMark-ET parameters, for example emission probabilities of intron related states, does not depend on repeat masking. On the other hand, some groups of parameters (e.g. models of non-coding regions) derived from initially unlabeled data in unsupervised fashion show dependence from masking. Still, the effect of repeat masking prior to training on the gene prediction accuracy of GeneMark-ET is not very significant.

Finally, we want to emphasize that the new method, GeneMark-ET, is about the integration of RNA-Seq read mapping into the *training* to improve estimation of parameters of the gene prediction algorithm. How useful is incorporating RNA-Seq mapping into the *prediction* step is yet another question. A full RNA transcript, if mapped to genome without errors, would identify a gene with high accuracy. NGS technology produces short RNA-Seq reads that must be assembled into transcripts. Recent assessment of the quality of transcript assembly from RNA-Seq reads (8) has shown that there is still significant room for improvement. For a given genome, a significant fraction of transcript assemblies carries errors (e.g. Figure 5 in Steijgler *et al*., 2013 even for one of the best tools, Cufflinks (28), reports errors in about 60% of transcripts assemblies for *H. sapiens*, about 50% for *D. melanogaster* and about 40% for *C. elegans*). This assessment makes it also clear that creation of a reliable training set for an *ab initio* algorithm by using genes mapped from assembled RNA-Seq transcripts is a non-trivial task. The errors in transcript assemblies will make the set corrupted to a significant degree.

The semi-supervised approach proposed here bypasses the task of RNA-Seq reads ‘assembly’. Also, for the purpose of selecting ‘anchored splice sites’ the algorithm treats RNA-Seq reads equally regardless of their source. This approach is likely to produce unbiased parameters; also it shows robustness to variations in volumes of RNA-Seq. Notably, use of anchored splice sites eliminates false positive outcomes in RNA-Seq read mapping and discriminates mapped introns situated in untranslated regions (UTRs) and RNA genes.

Since GeneMark-ET runs less iteration to convergence than GeneMark-ES (Figure 4) the running time of GeneMark-ET is shorter. In absolute terms, for *D. melanogaster* 100 Mb genomic sequence the GeneMark-ET run to convergence (five runs of the Viterbi algorithm) takes three hours on a single 3GHz CPU. A node with several CPUs, not to say about a cluster with several nodes, reduces the running time to dozen minutes (same is true for GeneMark-ES). However, to run GeneMark-ET an additional time has to be taken by the algorithm aligning RNA-Seq reads.

In general, unsupervised training algorithms that we pioneered for compact eukaryotic genomes (up to 300 Mb in length) are fast and sufficiently accurate (5,6). They provide the advantage of accelerating the annotation project and getting information on new genes and proteins in a short time. Unsupervised training algorithms are likely to be more accurate than algorithm with supervised training if the sets of validated genes available for supervised training are small; with accumulation of validated genes (the process that may take months, even years) algorithms with supervised training have no reason to produce less accurate gene predictions than algorithms with unsupervised training.

## CONCLUSION

We describe the new algorithm, GeneMark-ET that employs semi-supervised training to estimate parameters of the hidden semi-Markov model. We introduce a novel training approach that augments developed earlier unsupervised training technique by use of readily available unassembled RNA-Seq reads. We show that RNA-Seq read alignments incorporated into the GeneMark -ET procedure of parameter estimation improves the accuracy of gene prediction.

## AVAILABILITY

The GeneMark-ET software can be downloaded from topaz.gatech.edu/license_download.cgi. An RNA-Seq read aligner is supposed to be installed according to its own instructions as a separate tool.

## SUPPLEMENTARY DATA

Supplementary Data are available at NAR Online.

SUPPLEMENTARY DATA
