# Supplementary material for: Integration of mapped RNA-Seq reads into automatic training of eukaryotic gene finding algorithm
Source: Nucleic Acids Res. 2014 Jul 2;42(15):e119. doi: 10.1093/nar/gku557 (PMC4150757; doi:10.1093/nar/gku557)
Supplement: SUPPLEMENTARY DATA [file supp_42_15_e119__index.html]

Integration of mapped RNA-Seq reads into automatic training of eukaryotic gene finding algorithm — Integration of mapped RNA-Seq reads into automatic training of eukaryotic gene finding algorithm — SUPPLEMENTARY DATA 

# Integration of mapped RNA-Seq reads into automatic training of eukaryotic gene finding algorithm

## SUPPLEMENTARY DATA

**Files in this Data Supplement:**

- Supplementary Table
