# Supplementary material for: Integration of mapped RNA-Seq reads into automatic training of eukaryotic gene finding algorithm
Source: Nucleic Acids Res. 2014 Jul 2;42(15):e119. doi: 10.1093/nar/gku557 (PMC4150757; doi:10.1093/nar/gku557)
Supplement: SUPPLEMENTARY DATA [file supp_gku557_nar-02839-met-n-2013-File002.docx]

**Integration of Mapped RNA-Seq Reads into Automatic Training
of Eukaryotic Gene Finding Algorithm**

Alexandre Lomsadze^1^, Paul D. Burns^1^ and Mark Borodovsky^1,2,3,*^

^1^ Joint Georgia Tech and Emory Wallace H. Coulter Department of Biomedical Engineering, Atlanta, GA, USA

^2^ School of Computational Science & Engineering, Georgia Tech, Atlanta, GA, USA

^3^ Department of Bioinformatics, Moscow Institute of Physics and Technology, Moscow, Russia

* - corresponding author

**Supplementary Data**

**Table S1**. Performance of GeneMark-ET working with different RNA-seq alignment programs is shown for *D. melanogaster* and *A. gambiae*. The results show robustness of the algorithm with respect to use of one or another aligner. Still, in case of *D. melanogaster* UnSplicer produced better support for the GeneMark-ET training, while for *A. gambiae* TopHat shows better performance.


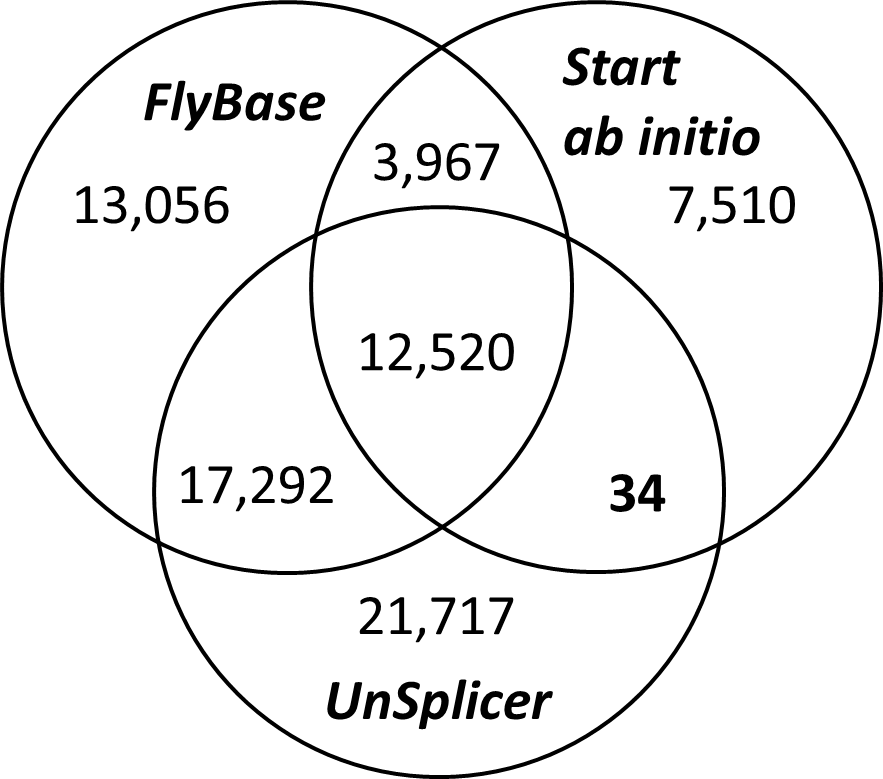


**Figure S1.** Comparison of introns annotated in FlyBase release 48 with the whole set of introns mapped by UnSplicer (with scores from 0 to 1) and with the set of introns predicted by the *ab initio* algorithm GeneMark.hmm at the first iteration of GeneMark-ET. Only 34 (0.3%) of introns simultaneously supported by UnSplicer and by the *ab initio* gene finder are not included into FlyBase annotation. Intron sets annotated in FlyBase and mapped by UnSplicer include introns located in untranslated regions (UTRs) as well as introns from genes with alternatively spliced isoforms.
